# United States Emergency Department Use of Medications with Pharmacogenetic Recommendations

**DOI:** 10.5811/westjem.2021.5.51248

**Published:** 2021-09-23

**Authors:** Alexander T. Limkakeng, Pratik Manandhar, Alaatin Erkanli, Stephanie A. Eucker, Adam Root, Deepak Voora

**Affiliations:** * Duke University School of Medicine, Division of Emergency Medicine, Durham, North Carolina; † Duke University School of Medicine, Department of Biostatistics and Bioinformatics, Durham, North Carolina; ‡ Duke University School of Medicine, Department of Orthopedics, Durham, North Carolina; § Duke University Hospital, Department of Pharmacy, Durham, North Carolina; ¶ Duke University School of Medicine, Division of Cardiology, Durham, North Carolina

## Abstract

**Introduction:**

Emergency departments (ED) use many medications with a range of therapeutic efficacy and potential significant side effects, and many medications have dosage adjustment recommendations based on the patient’s specific genotype. How frequently medications with such pharmaco-genetic recommendations are used in United States (US) EDs has not been studied.

**Methods:**

We conducted a cross-sectional analysis of the 2010–2015 National Hospital Ambulatory Medical Care Survey (NHAMCS). We reported the proportion of ED visits in which at least one medication with Clinical Pharmacogenetics Implementation Consortium (CPIC) recommendation of Level A or B evidence was ordered. Secondary comparisons included distributions and 95% confidence intervals of age, gender, race/ethnicity, ED disposition, geographical region, immediacy, and insurance status between all ED visits and those involving a CPIC medication.

**Results:**

From 165,155 entries representing 805,726,000 US ED visits in the 2010–2015 NHAMCS, 148,243,000 ED visits (18.4%) led to orders of CPIC medications. The most common CPIC medication was tramadol (6.3%). Visits involving CPIC medications had higher proportions of patients who were female, had private insurance and self-pay, and were discharged from the ED. They also involved lower proportions of patients with Medicare and Medicaid.

**Conclusion:**

Almost one fifth of US ED visits involve a medication with a pharmacogenetic recommendation that may impact the efficacy and toxicity for individual patients. While direct application of genotyping is still in development, it is important for emergency care providers to understand and support this technology given its potential to improve individualized, patient-centered care.

## INTRODUCTION

Drug side effects, toxicity, and limited efficacy are common reasons for treatment failure and non-adherence and can lead to suboptimal outcomes.^1^ This can be particularly problematic from the emergency department (ED) where a brief interaction prevents optimal tailoring and adjustments of a patient’s medication regimen. One area that holds promise for potentially improving initial choice of treatment is pharmacogenetics. Pharmacogenetics refers to the way in which one or a number of genes influence drug effects. Collectively the study of these relationships comprises pharmacogenomics, the broader study of interactions between numerous genes across the whole genome and drug activity. These genetically determined interactions contribute to the observed variability in different patients’ responses to a given drug.

The potential improvement in treatment efficacy and decrease in medication-related morbidity has led the United States Food and Drug Administration to endorse many pharmacogenetic recommendations, ie, altering the dose or choosing an alternate medication for a specific indication based on the patient’s genotype. For example, the CYP2D6 gene has numerous alleles with a wide range of function, which can lead to phenotypes ranging from poor to ultrarapid metabolizers of opioids.^2^ Up to 28% of patients in some regions of Africa were found to have the ultrarapid metabolizer phenotype for CYP2D6,^3^ for which it is recommended to reduce doses of common ED medications such as tramadol, ondansetron, or oxycodone to prevent serious side effects or toxicity.

Excitingly, the ability to apply pharmacogenetic information in the ED may be just on the horizon. Many commercial products allow patients to have their entire genetic data sequenced and downloaded in portable formats, and insurance carriers frequently reimburse for specific genotype tests. This could enable any provider to review their data and provide pharmacogenetic-guided drug selection.^4^ Some healthcare systems are already screening and making available to their network providers relevant pharmacogenetic genotypes to help guide clinical care. Once a patient’s relevant genotype has been determined, this information can easily be stored in electronic health records (EHR) and used for actionable guidance in real time, similar to existing pop-up warnings for allergic drug reactions.^5^,^6^

The Clinical Pharmacogenetics Implementation Consortium (CPIC) guidelines^7^ catalog known pharmacogenetic recommendations into evidence-based recommendations for specific gene–drug pairs. The use of these guidelines can lead to increased efficacy or decreased toxicity from a number of commonly prescribed medications. Therefore, an important first step toward understanding the potential benefit for the application of these guidelines in the ED is to characterize the types and frequencies of medications with pharmacogenetic recommendations that are ordered in EDs in the US. This information could shed light on the potential impact of pharmacogenetic guidance on patient outcomes in the ED.

The US Centers for Disease Control and Prevention National Hospital Ambulatory Medical Care Survey (NHAMCS) allows researchers to calculate nationalized estimates of US ED visit characteristics, including medications ordered and prescribed. We conducted a cross-sectional study using the NHAMCS to determine what proportion of US ED visits included orders for medications with pharmacogenetic recommendations. Secondarily, we sought to determine patient-level characteristics associated with these visits to determine whether there are high-yield subgroups that might benefit from pharmacogenetic genotyping.

**Population Health Research Capsule**

What do we already know about this issue?

*Emergency departments (ED) use medications with different efficacy and side effect profiles. Many drugs have recommendations based on the patient’s specific genotype.*

What was the research question?

*How frequently are medications with pharmacogenetic recommendations used in United States’ (US) EDs?*

What was the major finding of the study?

*Over 18% of US ED visits involve a medication with a pharmacogenetic recommendation that may impact efficacy or toxicity.*

How does this improve population health?

*Systems to support pharmacogenetic recommendations hold promise for improving emergency care through more targeted therapies with better efficacy.*

## MATERIALS AND METHODS

### Study Design and Setting

We analyzed the NHAMCS 2010–2015 datasets. The NHAMCS uses a multi-staged probability sample design to collect a nationally representative sample of all US ambulatory care visits, excluding federal and military hospitals. We restricted our analysis to ED visits only. This study was exempted from full board review by the Duke Health Institutional Review Board.

### Methods and Measurements

The NHAMCS survey methods have been described in detail previously.^8^ Briefly, hospitals are selected for discrete visit sampling through 112 geographic primary sampling units, with approximately 480 hospitals being surveyed. The NHAMCS collects demographic data, hospital characteristics, medications ordered or prescribed for each visit, and the final ED disposition.

### Data Collection and Processing

We downloaded NHAMCS data for 2010–2015 in November 2018. All data analysis was carried out using SAS 9.4 (SAS Institute, Cary, NC). We extracted the following variables from NHAMCS ED visits: age; race/ethnicity; gender; insurance status of the patient; medications ordered; hospital characteristics (geographic location and metropolitan area); disposition from the ED (admission, discharge, transfer); and year of visit. The CPIC compiles a list of medications with pharmacogenetic recommendations and grades the level of evidence (with “A” indicating the highest level of evidence). In May 2019, the lead author reviewed CPIC’s list of medications with Level A or B evidence and removed those that are not commonly prescribed in EDs. We studied the remaining 21 medications and report those that were involved in at least 0.1% of ED visits nationally (Table S1).

### Outcome Measures

Our primary outcome measure was percentage of ED visits in which a CPIC medication was ordered.

### Data Analysis

We calculated raw percentages for demographics, hospital characteristics, and medications. National-level estimates were derived using the weights assigned by the National Center for Health Statistics for each visit. Weights are included in the dataset for each survey visit to account for selection probabilities, nonresponse, population ratio adjustment, and weight smoothing. Patients were sorted into subgroups for analysis. Our first subanalysis divided patients by the number of CPIC medications they were prescribed during their ED visit. We then compared the distributions of age, gender, race/ethnicity, disposition, geographical region, immediacy, and insurance status between the overall ED population and those patients receiving a CPIC medication, using 95% confidence intervals.

## RESULTS

During 2010–2015, there were 165,155 entries representing 805,726,000 US ED visits in the NHAMCS. Among these, there were 148,243,000 (18.4%) ED visits in which CPIC medications were ordered. The percentage of ED visits involving a CPIC medication increased from 15.7% in 2010 to 21.3% in 2015 (Figure).

The demographics of ED patients overall and those with visits involving CPIC medications are summarized in Table 1. Visits involving CPIC medications had significantly higher proportions of female patients and dispositions of discharge from the ED but significantly lower proportions of patients with Medicare and Medicaid. There were minimal differences between geographical areas or hospital-based characteristics.

The percentage of ED visits involving a CPIC medication, along with the level of evidence, are presented in Table 2. The most common CPIC medication was tramadol (6.3%), followed by ondansetron (4.0%) and oxycodone (3.5%). Table 3 lists gene–drug pairings of commonly ordered or prescribed medications in the ED along with prevalence of affected genotypes and actionable recommendations with rationale.

## DISCUSSION

Emergency departments in the US administer a wide range of medications, many of which have pharmaco-genetic recommendations to adjust the dose or choice of medication based on patients’ genotypes to improve treatment efficacy and reduce toxicity and side effects. In this study we identified a sizeable proportion of ED visits, from 15–20%, involving the ordering or prescribing of a medication with a CPIC pharmacogenetic recommendation based on a high level of evidence. Over the six-year period studied, the number of gene–drug pairs with a high level of evidence has grown and is expected to continue to grow with continued research in this field. Thus, pharmacogenetics is expected to become increasingly relevant to emergency medicine as the genotypes contributing to the clinically observed variation in medication response phenotypes become elucidated.

The potential impact of pharmacogenetic-guided therapy in a variety of other healthcare settings has been described.^9^ A trial of CYP2D6-guided pain treatment suggested improved pain control from opioids for patients with chronic pain.^10^ Acute pain could similarly benefit from more targeted use of medications for more effective pain control in the ED and mitigation of opioid use disorder development.^11^,^12^ To our knowledge, ours is the first study focused on EDs, in which we found the top three most frequently prescribed CPIC medications are commonly used for treating pain and nausea. Poor pain control is still one of the most frequently cited reasons for lack of patient satisfaction with ED care, and a common reason for poor post-ED discharge outcomes.^13^ Medication side effects are an additional major patient complaint.^12^ Accordingly, patient-centered care in EDs would benefit from systems to support pharmacogenetically guided treatment to improve treatment efficacy, medication tolerability, and patient-oriented outcomes.

Although genotype testing is not currently readily available in a platform that can be performed during an ED visit, completion of genetic or genomic testing by an outpatient provider prior to a patient’s ED visit could make it available for informing more acute medical care. For example, direct-to-consumer genetic testing companies offer 12 pharmacogenetic tests to their United Kingdom customers.^14^,^15^ There are also targeted laboratory blood test panels that can identify common genotypes associated with pharmacogenetic recommendations.^16^ Existing EHR technologies could enable uploading of this genotype data to the patient’s medical record, allowing access to this data and embedded decision-support tools to inform emergency care providers of the pharmacogenetic recommendations associated with the patient’s genotype. Given the rapid expansion of EHR systems including health information exchanges, it may soon be feasible for emergency physicians to access previously conducted genetic testing results in an actionable way.

## LIMITATIONS

In the current study, we did not know the specific genotypes of the patients being studied and were not able to determine whether optimal therapies were given nor what the patient-level effects were. Furthermore, since we retrospectively analyzed this data, we were unable to determine whether other factors influenced drug selection, such as prior medication use or drug-drug interactions. Therefore, the degree of direct clinical benefit from pharmacogenetically guided therapy remains unknown, particularly in an acute setting. However, recent systematic reviews on the wide variability of patient response and large, side-effect profiles of common ED medications suggest that a large number of patients have relevant pharmacogenetics that remain to be elucidated and used for clinical benefit.^12^ Our conclusions are based on data from 2010–2015, and there have been efforts to decrease opioid medication prescriptions since that time. Therefore, estimates of ED visits including these medications may have changed.

## CONCLUSION

A significant proportion of ED patients are prescribed medications for which there are pharmacogenetic recommendations. Systems to identify such patients and to support clinicians toward more targeted therapies with better efficacy and side-effect profiles hold promise for improving emergency care. Future work should identify the prevalence of specific genotypes and corresponding phenotypes relevant to pharmacogenetic guidance in US EDs, develop feasible systems for testing, storing and accessing patient genetic phenotypes, and determine the degree of clinical benefit that might be derived from pharmacogenetically guided therapy in the ED.

## Supplementary Information



## Figures and Tables

**Figure:**
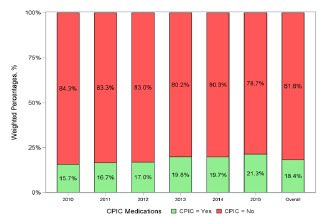
Rates of CPIC medications prescribed by year.

**Table 1:** Demographics and comparison of visits with a CPIC medication.

Variable	All ED Visits, 2010–2015		Visits in which a CPIC medications was ordered (2010–2015)	
	
	Weighted estimate (%) (95% CI)	Weighted patient # (in 1000s)	Weighted estimate (%) (95% CI)	Weighted patient # (in 1000s)
Patient age in years		805,726		148,243
Median	33.8 (33.0, 34.6)		36.8 (36.0, 37.6)	
Quartile 1	18.8 (18.0, 19.6)		24.1 (23.5, 24.6)	
Quartile 3	53.9 (53.1, 54.6)			
Race/ethnicity (RACER and ETHIM combined)				
Non-Hispanic White	59.2 (57.1, 61.3)	476,805	61.3 (58.9, 63.6)	90,805
Non-Hispanic Black	22.4 (20.2, 24.5)	180,130	21.6 (19.3, 23.9)	31,970
Hispanic	15.5 (13.9, 17.1)	124,909	14.5 (12.8, 16.3)	21,534
Non-Hispanic Other	3.0 (2.5, 3.4)	23,882	2.7 (2.2, 3.1)	3,933
Patient Sex				
Female	55.3 (54.8, 55.7)	445,253	57.5 (56.7, 58.4)	85,288
Expected primary source of payment (based on hierarchy)				
Private insurance	28.6 (27.5, 29.6)	230,145	31.9 (30.3, 33.4)	47,256
Medicare	18.2 (17.5, 18.9)	146,598	16.4 (15.4, 17.4)	24,308
Medicaid or CHIP	28.3 (27.0, 29.5)	227,873	24.1 (22.6, 25.5)	35,681
Self-pay	13.1 (12.3, 13.8)	105,473	15.7 (14.7, 16.7)	23,285
Unknown	6.1 (5.0, 7.1)	48,878	5.6 (4.5, 6.7)	8,300
Worker’s compensation	0.9 (0.8, 0.9)	6,857	1.0 (0.9, 1.2)	1,518
All sources of payment are blank	1.3 (0.9, 1.7)	10,470	1.3 (0.8, 1.7)	1,865
No charge/Charity	0.9 (0.6, 1.2)	7,113	1.2 (0.7, 1.6)	1,760
Other	2.8 (2.4, 3.2)	22,320	2.9 (2.4, 3.4)	4,270
Immediacy with which patient should be seen				
Immediate	0.8 (0.6, 0.9)	6,369	0.7 (0.5, 0.8)	1,030
Emergent	8.3 (7.6, 8.9)	66,615	7.0 (6.3, 7.7)	10,385
Urgent	35.9 (34.2, 37.6)	289,416	39.1 (36.9, 41.3)	57,927
Semi-urgent	28.8 (27.4, 30.2)	231,851	28.1 (26.4, 29.8)	41,696
Nonurgent	5.7 (5.1, 6.4)	46,234	4.5 (3.7, 5.3)	6,666
Visit occurred in ED that does not conduct nursing triage	2.5 (1.7, 3.3)	20,131	2.2 (1.4, 2.9)	3,194
Discharged from the ED				
Yes	89.6 (88.9, 90.4)	722,120	91.6 (90.5, 92.7)	135,827
Admit to this hospital				
Yes	10.4 (9.6, 11.1)	83,607	8.4 (7.3, 9.5)	12,416
Metropolitan statistical area status				
MSA (Metropolitan Statistical Area)	83.5 (77.9, 89.2)	563,706	82.5 (76.2, 88.8)	103,963
Non-MSA	16.5 (10.8, 22.1)	111,151	17.5 (11.2, 23.8)	22,033
Geographic region				
Northeast	17.5 (14.7, 20.3)	140,858	16.1 (12.6, 19.7)	23,887
Midwest	23.2 (19.7, 26.7)	187,086	22.7 (18.7, 26.6)	33,617
South	38.5 (34.4, 42.6)	310,329	40.3 (35.3, 45.4)	59,792
West	20.8 (17.7, 23.9)	167,453	20.9 (16.8, 24.9)	30,946

*ED*, emergency department; *CPIC*, Clinical Pharmacogenetics Implementation Consortium; *CI*, confidence interval; *MSA*, Metropolitan Statistical Area; *CHIP*, Children’s Health Insurance Program.

**Table 2:** Rates of visits by common emergency department CPIC medications.

Medication (Gene)	2019 CPIC evidence level*	2020 CPIC evidence level*	Overall	

			Weighted patient # (in 1000s)	Estimate (95% CI)
Any CPIC Medications (Gene)			148,243	18.4% (17.6%, 19.2%)
Tramadol (CYPD2D6)	A	A	50,575	6.3% (5.9%, 6.6%)
Ondansetron (CYP2D6)	A	A	32,223	4.0% (3.6%, 4.4%)
Oxycodone (CYP2D6)	A	C	27,847	3.5% (3.0%, 3.9%)
Lidocaine (G6PD)	B	B/C	24,336	3.0% (2.8%, 3.2%)
Codeine (CYP2D6)	A	A	8,381	1.0% (0.9%, 1.1%)
Omeprazole (CYP2C19)	B	A	4,526	0.6% (0.5%, 0.6%)
Pantoprazole (CYP2C19)	B	A	4,241	0.5% (0.4%, 0.6%)
Ciprofloxacin(G6PD)	B	B	4,147	0.5% (0.4%, 0.6%)
Sulfamethoxazole/Trimethoprim (G6PD, NAT2)	B	B	2,650	0.3% (0.3%, 0.4%)
Erythromycin (G6PD)	B	(removed)	2,576	0.3% (0.3%, 0.4%)
Levofloxacin (G6PD)	B	(removed)	2,563	0.3% (0.3%, 0.4%)
Phenytoin (CYP2C9, HLA-B, SCN1A)	A	A, A, B	2,195	0.3% (0.2%, 0.3%)
Divalproex Sodium (POLG)	B	A/B	1,850	0.2% (0.2%, 0.3%)
Carbamazepine (HLA-A, HLA-B, SCN1A)	A	A, B	1,759	0.2% (0.2%, 0.3%)
Valproic Acid (POLG, ABL2, ASL, ASS1, CPS1, NAGS, OTC)	B	A/B, B	1,734	0.2% (0.2%, 0.3%)
Warfarin (CYP4F2, CYP2C9, VKORC1)	A	A	867	0.1% (0.1%, 0.1%)
Nitrofurantoin (G6PD)	B	B	809	0.1% (0.1%, 0.1%)
Clopidogrel (CYP2C19)	A	A	588	0.1% (0.0%, 0.1%)
Succinylcholine (RYR1, CACNA1S, BCHE)	A	A, B/C	584	0.1% (0.1%, 0.1%)
Moxifloxacin (G6PD)	B	B	449	0.1% (0.0%, 0.1%)
Dextromethorphan (CYP2D6)	B	B/C	226	0.0% (0.0%, 0.0%)

* CPIC assigns CPIC levels to gene/drug pairs. The levels (A, B, C, and D) represent the strength of level of evidence. Only those that have had sufficient in-depth review of evidence to provide definitive CPIC level assignments are published. Note that only CPIC level A and B gene/drug pairs have sufficient evidence for at least one prescribing action to be recommended. (https://cpicpgx.org/genes-drugs/) Accessed 5/6/19 and 12/11/20. Listed drugs may have more than one drug-gene pairing, only pairings with CPIC level A and/or B evidence are listed.

*CPIC*, Clinical Pharmacogenetics Implementation Consortium.

**Table 3:** Actionable pharmacogenetic guidance examples.

Medication	Gene pairing	Genotype prevalence*	Rationale	Action
Tramadol, Ondansetron, Oxycodone, Dextromethorphan	CYP2D6	Poor metabolizers 6–10% in European Caucasians; Approximately 30% of Asians intermediate metabolizers. Ultrarapid metabolizer up to 28% of North Africans, Ethiopians, and Arabs.^1^	Patients can be classified as ultra-rapid, intermediate, or poor metabolizers depending on specific genotype. This applies to all CYP2D6 gene-drug pairs.	Dose may need to be decreased (for ultrarapid) or increased (for intermediate). Alternative (non-CYP2D6-interacting) drug recommended for poor metabolizers.
Lidocaine, Fluoroquinolones**, Sulfamethoxazole/Trimethoprim, Erythromycin**, Nitrofurantoin	CYB5R1, CYB5R2, CYB5R3 and CYB5R4-G6PD		Patients with G6PD deficiency and carriers more susceptible to drug-induced methemoglobinemia	Use with caution.
Omeprazole/Pantoprazole	CYP2C19	3% Caucasians and 15 to 20% of Asians have reduced or absent CYP2C19 enzyme activity.^10^	Patients can be classified as ultra-rapid, intermediate, or poor metabolizers depending on specific CYP2C19 genotype.	Ultrarapid: Increased dose may be needed.
Phenytoin	CYP2C9, HLA-B, SCN1A	HLA-B*15:02 is most prevalent in Oceania and Asian populations, ranging from 1–10%. CYP2C9 poor intermediate metabolizers range from 25–75% prevalence.^11^	HLA-B*15:02 carrier associated Stevens Johnson Syndrome (SJS). Patients can be classified as ultra-rapid, intermediate, or poor metabolizers depending on specific CYP2C9 genotype.	Do not use in HLA-B*15:02. Intermediate, poor metabolizers: reduce initial dose
Divalproex Sodium, Valproic Acid	POLG		Specific genotypes predict risk of Valproate Sodium hepatic toxicity.^12^	Avoid carbamazepine in these genotypes.^13^
Carbamazepine	HLA-A, B, SCN1A	See above	HLA-B*15:02 carrier associated SJS. HLA-A*31:01allele is associated with a wider range of carbamazepine hypersensitivity reactions, including MPE, DRESS, and SJS/TEN.	Avoid carbamazepine in these genotypes.^13^
Warfarin	CYP2C9 ; CYP4F2; VKORC1	Allele Frequency ranges from 3.4–23.1.^14^	18 alleles have been associated with decreased enzyme activity. The nonsynonymous variant CYP4F2*3 (c.1297G>A; p.Val433Met; rs2108622) was first shown to affect enzyme activity. A common variant upstream of VKORC1(c.-1639G>A,rs9923231) is significantly associated with warfarin sensitivity	Algorithm-based dosing.
Clopidogrel	CYP2C19	See above	CYP2C19*2 heterozygotes and homozygotes have reduced active clopidogrel metabolites and higher on-treatment platelet aggregation compared with *1 homozygotes.^15^	Intermediate, poor metabolizers: Alternative antiplatelet therapy
Succinylcholine	RYR1; CACNA1S, BCHE		Certain subtypes associated with malignant hyperthermia	Use alternative agent.

* Subpopulations cited in this column refer to people living in particular geographic areas or ancestries as reported by the cited references, not race/ethnicities. Race/ethnicity may not serve as proxies for genetic ancestry.

** CPIC guidelines for Erythromycin and Levofloxacin have subsequently been removed in 2020 based on new evidence.

*G6PD*, Glucose-6-Phosphate Dehydrogenase; *MPE*, maculopapular exanthema; *DRESS*, Drug reaction with eosinophilia and systemic symptoms; *SJS*, Stevens Johnson Syndrome; *TEN*, Toxic epidermal necrolysis.

## References

[1] Tamma PD, Avdic E, Li DX (2017). Association of adverse events with antibiotic use in hospitalized patients. JAMA Intern Med.

[2] Crews KR, Monte AA, Huddart R (2021). Clinical Pharmacogenetics Implementation Consortium Guideline for CYP2D6, OPRM1, and COMT Genotypes and Select Opioid Therapy. Clin Pharmacol Ther.

[3] Dean L, Kane M, Pratt VM (2012). Tramadol therapy and CYP2D6 genotype. Medical Genetics Summaries.

[4] Limkakeng AT, Monte AA, Kabrhel C (2016). Systematic molecular phenotyping: a path toward precision emergency medicine?. Acad Emerg Med.

[5] Caraballo PJ, Sutton JA, Giri J (2020). Integrating pharmacogenomics into the electronic health record by implementing genomic indicators. J Am Med Inform Assoc.

[6] Roden DM, Pulley JM, Basford MA (2008). Development of a large-scale de-identified DNA biobank to enable personalized medicine. Clin Pharmacol Ther.

[7] Relling MV, Klein TE (2011). CPIC: Clinical Pharmacogenetics Implementation Consortium of the Pharmacogenomics Research Network. Clin Pharmacol Ther.

[8] McCaig LF, Burt CW (2012). Understanding and interpreting the National Hospital Ambulatory Medical Care Survey: key questions and answers. Ann Emerg Med.

[9] Luzum JA, Pakyz RE, Elsey AR (2017). The Pharmacogenomics Research Network Translational Pharmacogenetics Program: outcomes and metrics of pharmacogenetic implementations across diverse healthcare systems. Clin Pharmacol Ther.

[10] Smith DM, Weitzel KW, Elsey AR (2019). CYP2D6-guided opioid therapy improves pain control in CYP2D6 intermediate and poor metabolizers: a pragmatic clinical trial. Genet Med.

[11] Collins FS, Koroshetz WJ, Volkow ND (2018). Helping to end addiction over the long-term: the research plan for the NIH HEAL Initiative. JAMA.

[12] Busse JW, Sadeghirad B, Oparin Y (2020). Management of acute pain from non-low back, musculoskeletal injuries: a systematic review and network meta-analysis of randomized trials. Ann Intern Med.

[13] Brown T, Shetty A, Zhao DF (2018). Association between pain control and patient satisfaction outcomes in the emergency department setting. Emerg Med Australas.

[14] Lu M, Lewis CM, Traylor M (2017). Pharmacogenetic testing through the direct-to-consumer genetic testing company 23andMe. BMC Med Genomics.

[15] Guinto J (2014). Why is this $99 home DNA kit causing such an uproar?. Genome.

[16] van der Wouden CH, van Rhenen MH, Jama WOM (2019). Development of the PGx-passport: a panel of actionable germline genetic variants for pre-emptive pharmacogenetic testing. Clin Pharmacol Ther.

