# Supplementary material for: United States Emergency Department Use of Medications with Pharmacogenetic Recommendations
Source: West J Emerg Med. 2021 Sep 23;22(6):1347–54. doi: 10.5811/westjem.2021.5.51248 (PMC8597689; doi:10.5811/westjem.2021.5.51248)
Supplement: Supplementary file 1 [file wjem-22-1347-s001.docx]

Supplemental Table 1. List of Medications and NHAMCS Codes Used

| **CPIC Medication List** | **NHAMCS Medication Code used:** |
| --- | --- |
| 1. Oxycodone | 1. ACETAMINOPHEN W/OXYCODONE 2. OXYCODONE ER 3. OXYCODONE 4. OXYCODONE HCL 5. OXYCODONE HCL & ACETAMINOPHEN 6. OXYCODONE W/ASPIRIN |
| 1. Ondansetron | 1. ONDANSETRON |
| 1. Tramadol | 1. TRAMADOL HCL 2. ACETAMINOPHEN-TRAMADOL 3. TRAMADOL |
| 1. Phenytoin | 1. PHENYTOIN SODIUM EXTENDED 2. PHENYTOIN 3. FOSPHENYTOIN |
| 1. Valproic acid | 1. VALPROIC ACID |
| 1. Divalproex sodium | 1. DIVALPROEX SODIUM |
| 1. Ciprofloxacin | 1. CIPROFLOXACIN EYE SOLUTION 2. CIPROFLOXACIN-DEXAMETHASONE 3. CIPROFLOXACIN |
| 1. Levofloxacin | 1. LEVOFLOXACIN |
| 1. Moxifloxacin | 1. MOXIFLOXACIN |
| 1. Sulfamethoxazole/   trimethoprim | 1. SULFAMETHOXAZOLE-TRIMETHOPRIM 2. SULFAMETHOXAZOLE 3. TRIMETHOPRIM/SULFAMETHOXAZOLE |
| 1. Nitrofurantoin | 1. NITROFURANTOIN 2. NITROFURANTOIN MACROCRYSTALS |
| 1. Erythromycin | 1. ERYTHROMYCIN BASE-NEOMYCIN 2. ERYTHROMYCIN BENZOYL PEROXIDE 3. ERYTHROMYCIN 4. ERYTHROMYCIN OPHTHALMIC 5. ERYTHROMYCIN ETHYLSUCCINATE 6. ERYTHROMYCIN ESTOLATE |
| 1. Omepraxole | 1. OMEPRAZOLE-SODIUM BICARBONATE 2. OMEPRAZOLE |
| 1. Pantroprazole | 1. PANTOPRAZOLE SODIUM 2. PANTOPRAZOLE |
| 1. Succinylcholine | 1. SUCCINYLCHOLINE 2. SUCCINYLCHOLINE |
| 1. Carbamazepine | 1. CARBAMAZEPINE |
| 1. Clopidogrel | 1. CLOPIDOGREL |
| 1. Warfarin | 1. WARFARIN |
| 1. Lidocaine | 1. MAALOX W/ VISCOUS LIDOCAINE 2. KENALOG/LIDOCAINE 3. LIDOCAINE W/MARCAINE 4. DEPO-MEDROL W/ LIDOCAINE 5. LIDOCAINE/PRILOCAINE 6. LIDOCAINE/MAALOX/BENADRYL 7. ROCEPHIN W/LIDOCAINE 8. LIDOCAINE/MAALOX/NYSTATIN 9. LIDOCAINE/TETRACAINE 10. LIDOCAINE 11. LIDOCAINE HCL 12. LIDOCAINE HCL W/EPINEPHRINE 13. LIDOCAINE HCL VISCOUS 14. BUFFERED LIDOCAINE 15. LIDOCAINE BICARBONATE 16. LIDOCAINE/EPINEPHRINE/TETRACAINE |
| 1. Codeine | 1. ACETAMINOPHEN W/CODEINE 2. BUTALBITAL W/CODEINE 3. CODEINE 4. CODEINE PHOSPHATE 5. CODEINE SULFATE 6. DIHYDROCODEINE 7. ESGIC W/CODEINE 8. FIORINAL W/CODEINE 9. GUAIFENESIN W/CODEINE 10. GUIATUSSIN W/CODEINE 11. PHENERGAN W/CODEINE 12. PHENERGAN W/CODEINE 13. PROMETHAZINE HCL W/CODEINE 14. PROMETHAZINE VC W/CODEINE 15. PROMETHAZINE W/CODEINE 16. TYLENOL W/CODEINE 17. GUAIFENESIN/P-EPHED/CODEINE 18. FIORICET/ CODEINE |
| 1. Dextromethorphan | 1. DEXTROMETHORPHAN/GUAIFENSIN 2. DEXTROMETHORPHAN 3. APAP/DEXTROMETHORPHAN 4. DEXTROMETHORPHAN/PROMETHAZINE 5. GUAIFENESIN & DEXTROMETHORPHAN |
